# 4318 A Qualitative Study of Men’s Abortion Attitudes in Restrictive States

**DOI:** 10.1017/cts.2020.249

**Published:** 2020-07-29

**Authors:** Chris Ahlbach, Lori Freedman, Jennifer Kerns

**Affiliations:** 1 University of California San Francisco

## Abstract

OBJECTIVES/GOALS: Despite its critical importance in reproductive health, access to abortion care continues to be impeded by laws grounded in religious, political, or other ideologies. We will characterize abortion attitudes among US men who live in areas with restrictive abortion laws using qualitative methods. METHODS/STUDY POPULATION: We will use a semi-structured interview guide to elicit men’s attitudes about abortion, characterized within moral, legal, religious, political, and other domains. Inclusion criteria include English-speaking cisgender men, ages 18 to 65 who live in states with the most restrictive abortion laws as defined by the Guttmacher Institute. We will recruit participants through Facebook ads and interviews will continue until theoretic sufficiency. Using an inductive thematic analysis approach, transcripts will be coded for emergent themes by two researchers independently in QRS NVivo 12.0, with concurrent refinement of themes as interviews are completed. RESULTS/ANTICIPATED RESULTS: We will elucidate emergent themes regarding men’s abortion attitudes which could include how men think of abortion as a medical, moral, or personal reality, why they do or do not support abortion provision, among many other possibilities. We anticipate that researchers can use the data obtained from this study to begin to build a conceptual framework of abortion attitudes among US men who lives in restrictive states. DISCUSSION/SIGNIFICANCE OF IMPACT: This study will fill an important gap in the literature by qualitatively characterizing abortion attitudes among a population that has political influence on abortion access. Results can inform policy and advocacy campaigns aimed at shifting public abortion attitudes towards increased acceptance.

